# Prevalence and factors associated with arterial hypertension in a Brazilian rural working population

**DOI:** 10.6061/clinics/2020/e1603

**Published:** 2020-08-06

**Authors:** Luciane Bresciani Salaroli, Monica Cattafesta, Glenda Blaser Petarli, Sarah Aparecida Vieira Ribeiro, Ana Cristina de Oliveira Soares, Eliana Zandonade, Olívia Maria de Paula Alves Bezerra, José Geraldo Mill

**Affiliations:** I Programa de Pos-graduacao em Saude Coletiva, Universidade Federal do Espirito Santo, Vitoria, ES, BR.; II Programa de Pos-graduacao em Nutricao e Saude, Universidade Federal do Espirito Santo, Vitoria, ES, BR.; III Departamento de Nutricao e Saude, Universidade Federal de Vicosa, Vicosa, MG, BR.; IV Departamento de Medicina de Familia, Saude Mental e Coletiva, Universidade Federal de Ouro Preto, Ouro Preto, MG, BR.; V Programa de Pos-graduacao em Fisiologia, Universidade Federal do Espirito Santo, Vitoria, ES, BR.

**Keywords:** Hypertension, Prevalence, Rural Population, Farmers, Occupational Health

## Abstract

**OBJECTIVES:**

To determine the prevalence of hypertension and associated factors in farmers in a rural region of Brazil.

**METHODS:**

A cross-sectional study was conducted involving a sample of 790 farmers who were residents of Espírito Santo, Brazil.

**RESULTS:**

The prevalence of hypertension was 35.8% (95%CI: 32.5-39.1%, n=283); however, it was higher in men (36.6%, n=151, *p<*0.001) and in those with excess weight (48.9%, n=197, *p<*0.001). Of the 283 hypertensive patients, 125 (44.2%) did not use antihypertensive drugs. In men, lower level of schooling (*p=*0.004), working in the field for fewer daily hours (*p<*0.001), and having greater abdominal adiposity (*p=*0.039) were associated with the presence of increased blood pressure. In women, age (*p=*0.002), lower schooling (*p=*0.021), and increased central adiposity (*p=*0.003) were independent predictors of blood pressure.

**CONCLUSION:**

The highest prevalence of hypertension was observed in men, with elevated blood pressure being strongly associated with social and economic factors. In women, the association with the classic factors (age, increase in abdominal adiposity, and low schooling) was stronger. In addition, most hypertensive patients are not adequately diagnosed or treated.

## INTRODUCTION

Hypertension is a multifactorial disease of complex nature since its occurrence depends on both genetic factors and lifestyle. Given its high prevalence and its relationship with cardiovascular morbidity and mortality, hypertension is a public health problem in all countries, regardless of the level of development (1). In Brazil, hypertension contributes, directly or indirectly, to approximately 50% of cardiovascular deaths, thus resulting in a high economic and social cost (2).

Despite these worrisome numbers, some studies have shown a reduction in the prevalence of hypertension in the last decades (2,3). However, the data analyzed in the aforementioned studies were largely obtained from urban populations. Given the great territorial extension and racial and social diversity, the incidence of hypertension is not uniformly distributed in Brazil. The National Health Survey (PNS) of 2013 (3), the only survey conducted with national coverage until now, revealed a 22.3% prevalence of hypertension, ranging from 13.2% in the state of Amazonas to 26.7% in the state of Rio de Janeiro.

Most studies on hypertension in Brazil were conducted in urban populations. However, approximately 20% of Brazilians live in rural areas and this population has been scarcely studied until now (4). Differences in lifestyle, schooling, access to information, and medical care may contribute to differences in the epidemiology of the disease. These factors must be described in different populations in order to subsidize public policies focused on specific population subgroups, such as people living in rural areas (5).

The municipality of Santa Maria de Jetibá, located in the mountainous region of the state of Espírito Santo, Brazil, has an economy strongly dependent on agricultural production, which derives from the work done by the families that reside in small rural properties. Most of the individuals descended from a German population (Pomeranians) that emigrated to Brazil in the late 19^th^ and early 20^th^ century (6). Cardiovascular mortality in this population is similar to the national average which is approximately 30% (7,8).

Thus, the objective of this study was to determine the prevalence of hypertension and its associated factors in farmers living in a rural Brazilian municipality.

## MATERIALS AND METHODS

### Study population

This is an epidemiological cross-sectional study that involved participants living in the municipality of Santa Maria de Jetibá, Brazil. The research population was made up of workers whose main source of income is agriculture. Participants between the ages of 18 and 59 years, who were not pregnant and were working actively for at least 6 months were included.

To define the sample universe, one list was built with the survey of the registration of individuals and families by the Community Health Agents (CHA), through the data by the Family Health Strategy teams. This register covers 100% of the eleven health regions of the municipality. At the time the study was conducted (September/2016), 4,018 families were enrolled; among them 7,287 were farmers. The participants were selected by stratified sampling, proportionality the number of families per health region, in order to respect proportionality among the eleven regions. In families with more than one eligible individual, only one individual was included, to avoid the interdependence of information. In case a participant refuses to participate or is absent during data collection, a new participant on the waiting list was called, respecting the sex and region of origin of the dropout.

Of the 806 invited farmers, 108 (13.4%) either declined or were absent during the data collection and were replaced with participants who were on the waiting list. At the end of the study, after excluding individuals with missing data or who were unable to complete all stages of data collection, the final sample consisted of 790 individuals (loss of 1.98%).

### Data collection

The data were collected by researchers trained for this purpose and by master's or doctoral students in the Nutrition and Public Health program, between December 2016 and April 2017. Participant information were obtained in an interview using a standardized questionnaire, followed by anthropometric and blood pressure measurements. From the data collected in the interviews, the following sociodemographic variables were obtained: sex (male/female), age group (decades), race/color self-referenced (grouped as “whites” and “nonwhites”), (“≤4 years,” “5-8 years,” or “>8 years”), ownership of the land where he/she works (“owner” and “nonowner,” this was used as a proxy for socioeconomic status), and weekly working hours (≤40 or >40 hours).

Regarding the lifestyle variables, alcohol consumption (consumer/non-consumer) and smoking (non-smoker, current smoker, and former smoker) were evaluated.

Body weight and height were obtained while participants were in the orthostatic position, barefoot, and wearing light clothing. The waist circumference (WC) was obtained with a tape measure (Sanny, Mod. TR-4010, São Bernardo do Campo, SP-Brazil) at the midpoint between the iliac crest and the lower rib. The anthropometric variables were classified according to guidelines established by the World Health Organization (WHO) (9) and categorized as “no abdominal obesity” for men with WC ≤94 cm and women with ≤80 cm and as “with abdominal obesity” for the other values. Body mass index (BMI) was classified according to WHO cut-off points (9) and grouped into “non-overweight” for BMI <25 kg/m^2^ and “overweight” for BMI ≥25 kg/m^2^. Body weight was measured using an Omron-514C^¯^ digital scale, with a capacity of 150 kg and a precision of 0.1 kg. The height was measured using a Sanny model ES-2060 portable stadiometer with an approximation of 0.1 mm. A venous blood sample was collected in the fasting state, and the glycemia was dichotomized as either “normal” (<100 mg/dL) or “altered” (≥100 mg/dL). For the logistic regression analysis, these variables were used in scale.

Blood pressure (BP) measurements were performed according to the procedures described in the VII Brazilian Hypertension Guideline (2). Three measurements were performed on the right arm, with the participant in the sitting position and after a rest period of at least 5 minutes, using a calibrated digital device (Omron HEM-7200, Shimogyo-ku, Kyoto-Japan) to obtain the systolic BP (SBP), diastolic BP (DBP), and heart rate. The first measure was discarded. The final value was determined as the mean of the second and third measurements. If the difference between them was ≥5 mmHg, a fourth measure was obtained and used as the final value. BP was classified, according to the guideline of the Brazilian Society of Hypertension (2), as: “normal” (SBP ≤120 mmHg and DBP ≤80 mmHg), “prehypertension” (SBP 121-139 mmHg and/or DBP 81-89 mmHg), and “hypertension” (SBP ≥140 mmHg and/or DBP ≥90 mmHg). The subjects who were using antihypertensive drugs, including diuretics, at the time of measurement were also considered hypertensive.

### Statistical analysis

For the sample size calculation, a prevalence of 50%, a sample error of 3.5%, a significance level of 95% were used, resulting in a minimum sample size of 708 farmers. All sample size calculations were performed using the EPIDAT software version 3.1.

The nominal variables were presented as absolute and relative frequencies. The associations between study variables and different BP ranges (normotensive, prehypertensive, and hypertensive) were evaluated using the Chi-square test. Quantitative variables were presented as mean±standard deviation.

Simple binary logistic regression was used to investigate factors associated with BP (hypertensive *versus* normotensive and prehypertensive), and the regression coefficient and 95% confidence interval (95%CI) were estimated. In the multiple binary logistic regression, the variables with a value of *p<*0.05 were included in the bivariate analysis. The analyses were performed in the IBM SPSS Statistics for Windows version 22.0 software (Armonk, NY: IBM Corp) and the statistical significance considered was *p<*0.05.

### Ethical precepts

The study was approved by the Research Ethics Committee (CEP) of the Health Sciences Center (CCS) of the Federal University of Espírito Santo under the number 1,856,331 (CAAE 52839116.3.0000.5060), according to the precepts of the Declaration of Helsinki. All participants signed the Free and Informed Consent Term (TCLE).

## RESULTS

**Figure 1:**
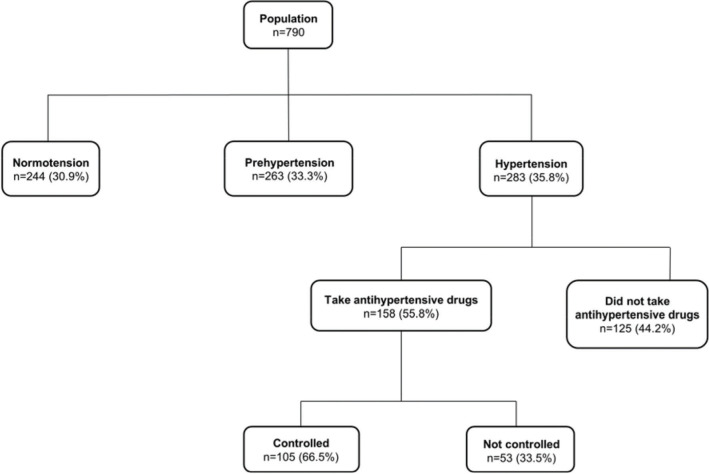
Flowchart of the characteristics of the population according to blood pressure.

In the sample of 790 farmers, 52.3% (n=413) were men. The mean age was 39.1±10.8 years (men, 39.5±10.9 years; women, 38.6±10.6 years; *p*>0.05). Normotension was observed in only 30.9% (95%CI: 27.7-34.1%) of the sample, while prehypertension was observed in 33.3% (95%CI: 30-36.6%). Hypertension was present in 35.8% (95%CI: 32.5-39.1%) of the sample, and among the 283 hypertensive patients, 125 (44.2%) did not take antihypertensive drugs (Figure 1). The prevalence of arterial hypertension was higher in men (36.6%, 95%CI: 33.2-40%) than in women (35.0%, 95%CI: 31.7-38.3%). Table 1 shows the sociodemographic and clinical characteristics of the normotensive, prehypertensive, and hypertensive groups. The distribution of groups was similar (*p*>0.05) according to race/color, land tenure, weekly workload, and alcohol consumption. The presence of hypertension was associated with increased age, low schooling, increased adiposity, and increased fasting glycemia. Although the prevalence of hypertension was similar between the sexes, the prevalence of prehypertension was significantly higher in men (43.8%, 95%CI: 40.3-47.3%) than in women (21.8%, 95%CI: 18.9-24.7%). Considering that the BP distribution was different between the sexes (Table 1), BP behavior was analyzed separately in men and women, considering the normal pressure and prehypertension *versus* hypertension of groups (Table 2).

We observed (Table 2) that the probability of men being hypertensive was negatively associated with schooling and weekly workload and positively associated with age and adiposity. The strength of the association (OR) was similar if obesity was inferred from the BMI or WC. Finally, increased glycemia was associated with a higher likelihood of hypertension in men, but not in women. The factors associated with the presence of hypertension in women were similar to those in men, except for race/color and indicators of obesity, since a significant positive association (*p<0.05*) was found with an increase in abdominal perimeter and absence association with BMI (general obesity index).

In the multiple regression analysis model (Table 3), the dependent variable was presence or absence of hypertension. For males, the independent variables associated were schooling >8 years [OR=0.257 (95%CI: 0.103-0.640); *p*=0.004], weekly workload >40 hours/week [OR=0.333 (95%CI: 0.187-0.596); *p*<0.001], and abdominal obesity [OR=1.947 (95%CI: 1.034-3.667); *p*=0.039]. For females, the independent variables associated were age [OR=2.260 (95%CI: 1.128-4.529), *p*=0.021 for those from 41-50 years old and OR=3.413 (95%CI: 1.568-7.432), *p=*0.002 for those >50 years old], schooling [OR=0.367 (95%CI: 0.176-0.763), *p=*0.007 for 5-8 years of schooling and OR=0.335 (95%CI: 0.132-0.851), *p=*0.021 for >8 years of schooling], and abdominal obesity [OR=2.531 (95%CI: 1.377-4.653); *p*=0.003]. The other variables were not statistical difference.

## DISCUSSION

In this study, a high prevalence of hypertension (35.8%) was detected in a random sample of farmers from a typical rural municipality in the Espírito Santo mountain region, which was well above the prevalence obtained from self-reports available in Brazil (20.9%) and in the rural Brazilian population (20.1%) (10). In the state of Espírito Santo, the prevalence of hypertension was 20.6% in the PNS-2013 (3). The value identified in this rural population was similar to that described for the urban population of Vitória (38.2%), the state capital (11).

We observed a strong inverse relationship between schooling and prevalence of hypertension. A similar relationship was not observed hypertension prevalence and land tenure, which can be considered a socioeconomic characterization index for people living in rural areas. In a study carried out in Zambia’s urban and rural areas, significant differences were found between the prevalence of hypertension among rural and urban inhabitants, and the prevalence of hypertension in the rural area was twice (46.9%) that observed in the urban area (22.9%). The authors concluded that such discrepancies may be due to different age structures among rural and urban populations or due to differences in lifestyles, such as daily caloric expenditure (greater use of muscles in rural areas) or alcohol consumption (12).

The results of this study also indicate that social aspects, such as schooling, are negatively associated with hypertension, corroborating the data of Bezerra et al. (13). Mariosa et al. (14) reported that social and environmental determinants, such as general living conditions, occupation, and access to health care, are relevant factors that can explain the variability in hypertension rates, since they are influenced by income and schooling.

In addition to the high prevalence of hypertension, it is noteworthy that the prevalence of prehypertension was much higher in men (43.8%) than in women (21.8%). The VII Brazilian Guideline of Hypertension makes it clear that prehypertensive individuals deserve as much clinical attention as hypertensive individuals, since BP should be monitored in order to eliminate risk factors and avoid the development of hypertension (2). Yano et al. (15), in a prospective cohort study with a mean follow-up period of 18.8 years, observed that young adults who had high BP during the early stages of hypertension were also at a higher risk of cardiovascular events in subsequent years compared to those with normal BP before the age of 40 years. This shows the importance of BP control in young individuals, even those in the prehypertensive stage. This is a major public health challenge worldwide due to its high prevalence and concomitant risk factors for cardiovascular and renal diseases (2,16).

The ethnic background of this group of farmers needs to be considered, since some genes may account for 5% to 9% of the interindividual variation in BP (17). Pena et al. (18), in a cohort of 931 individuals living in rural villages, reported that there may be a significant genetic influence on the cardiometabolic risk factors. By taking this into account, the Study of Health in Pomerania (SHIP-0 1997-2001), which involved 3,042 Pomeranians between the ages of 25-64 years who were living in Germany, reported a hypertension prevalence of 57% in men and 32% in women (19). However, in a recent study, no statistical difference was found relating to arterial hypertension and heart disease in a population of Germans and their descendants in a Brazilian region (20).

However, BP elevation may not only be due to genetic factors. Our results and those of Bezerra et al. (13) and Silva et al. (21), revealed a high prevalence of hypertension in communities with strong associated ethnic components, which suggests that among the Brazilian population, the risk of the development of hypertension is not solely determined by the race/color component. However, similar to genetic determinants, many other factors related to lifestyle influence BP elevation throughout life and, consequently, the occurrence of hypertension at some point in time. Variables related to dietary habits (high sodium and low potassium consumption) (22), excessive alcohol consumption (23), and low stress control (24) can also influence the development of hypertension. Studies conducted in Brazil, however, show that the prevalence of hypertension is higher among people with low socioeconomic status and educational backgrounds (25). However, the data analyzed in the aforementioned studies were obtained from urban populations.

Figure 1 shows that, although BP control using medication in hypertensive patients is possible, the result is poor, since controlled blood pressure was observed in only 53 out of 283 (18.7%) patients. This also occurred because a considerable proportion of hypertensive patients without medication (44.8%) did not know they had hypertension. As this rural population receives only primary health care, we can infer that one of the problems that need to be addressed is the low rate level of diagnosis of the disease and adherence to the treatment. These data confirm findings in other rural populations. Karmakar et al. (26) found that 51.8% of the hypertensive individuals studied in a rural community in West Bengal were not aware of their high BP, 52.9% of those diagnosed were not on pharmacological treatment, and only 8.8% had their BP under control. Nyaaba et al. (27) reported a substantial mismatch between community perceptions and the medical understanding of hypertension and its treatment, resulting both from the structural factors of society and collective traditions that shape beliefs and influence individual health behavior, socioeconomic factors, and adequate access to information. In addition, differences in demographic structures, household affluence, socioeconomic and occupational status, and patterns of consumption contribute to the rural-urban disparity in under-diagnosis and sub-medication rates (28).

Finally, the sample composition of this study is highlighted, because is representativeness of the studied population (n=790), reporting a sample loss of only 1.98% compared with the one calculated of safety margin of 20%. It is also worth noting that this number represents about 20% of all those eligible for the survey. Thus, this data demonstrates the robustness of our findings, enabling their extrapolation to the population evaluated. Another point to be considered is the internal consistency of the data, based on the analysis of the methodological criteria adopted, both during the sample selection stage, which shows that it followed quantitative parameters, respecting the principles of randomness and proportionality, and adoption of the method of data collection with the follow-up of validated protocols. However, the transversality of the data may lead to reverse causality in some findings and should be interpreted with caution, since individuals diagnosed with hypertension are more likely to change habits due to regular visits to the medical staff.

## CONCLUSIONS

We concluded that this population of farmers in a rural region of the state of Espírito Santo has a high prevalence of hypertension, which is above the national average and similar to that of urban regions. It is a community of Pomeranian descent whose socioeconomic and biological factors influence BP levels. The highest prevalence of hypertension and prehypertension were observed in men, and this increase depends on social, economic, labor, and biological factors. In women, classical factors such as schooling and abdominal adiposity predominated. In addition, most hypertensive patients are not adequately diagnosed or treated. Thus, it is necessary to improve the diagnosis and control of hypertension in this rural population in order to prevent its metabolic complications and reduce mortality due to cardiovascular diseases. It is also necessary to increase the population’s understanding of hypertension to increase adherence to treatment and preventive practices.

## AUTHOR CONTRIBUTIONS

All authors contributed to the design and construction of the manuscript. Salaroli LB, Cattafesta M, Petarli GB, Ribeiro SAV, Soares ACO, Zandonade E, Bezerra OMPA, and Mill JG contributed to the conception, planning, analysis, interpretation, and manuscript writing. All of the authors approved the final version of the manuscript.

## Figures and Tables

**Table 1:** Sociodemographic characteristics and nutritional status of farmers according to blood pressure classification.

	Normal BP 244 (30.9%)		Prehypertension 263 (33.3%)		Hypertension 283 (35.8%)		
Variables	n (%)	95%CI	n (%)	95%CI	n (%)	95%CI	*p* value*
**Sex**							
Male	81 (19.6)	16.8-22.4	181 (43.8)	40.3-47.3	151 (36.6)	33.2-40	**<0.001^a^,^b^**
Female	163 (43.2)	39.7-46.7	82 (21.8)	18.9-24.7	132 (35.0)	31.7-38.3	
**Age** (years)							
≤30	88 (41.3)	37.9-44.7	79 (37.1)	33.7-40.5	46 (21.6)	18.7-24.5	<0.001**^b^
31-40	82 (32.5)	29.2-35.8	93 (40.3)	36.9-43.7	56 (24.2)	21.2-27.2	
41-50	47 (24.1)	21.1-27.1	60 (30.8)	27.6-34	88 (45.1)	41.6-48.6	
>50	27 (17.9)	15.2-20.6	31 (20.5)	17.7-23.3	93 (61.6)	58.2-65	
**Race/color**							
White	218 (31.1)	27.9-34.3	231 (32.9)	29.6-36.2	253 (36.0)	32.7-39.3	0.810
Not white	26 (29.6)	26.4-32.8	32 (36.4)	33-39.8	30 (34.0)	30.7-37.3	
**Schooling** (years)							
≤4	142 (26.7)	23.6-29.8	166 (31.1)	27.9-34.3	225 (42.2)	38.8-45.6	<0.001**^b^
5-8	65 (37.6)	34.2-41	64 (37.0)	33.6-40.4	44 (25.4)	22.4-28.4	
>8	37 (44.0)	40.5-47.5	33 (39.3)	35.9-42.7	14 (16.7)	14.1-19.3	
**Possession of land**							
Owner	187 (30.2)	27-33.4	208 (34.1)	30.8-37.4	218 (35.7)	32.4-39	0.612
Non-owner	60 (33.3)	30-36.6	55 (30.6)	27.4-33.8	65 (36.1)	32.8-39.4	
**Workload** (hours/week)							
≤40	53 (32.7)	29.4-36	42 (25.9)	22.8-29	67 (41.4)	38-44.8	0.071
>40	191 (30.4)	27.2-33.6	221 (35.2)	31.9-38.5	216 (34.4)	31.1-37.7	
**Alcohol consumption**							
No	145 (32.7)	29.4-36	136 (30.7)	27.5-33.9	162 (36.6)	33.2-40	0.192
Yes	99 (28.5)	25.4-31.6	127 (36.6)	33.2-40	131 (34.9)	31.6-38.2	
**Smoking**							
Non-smoking	213 (32)	28.7-35.3	223 (33.6)	30.3-36.9	229 (32.4)	29.1-35.7	**0.031**
Current smoker	18 (29)	25.8-32.2	24 (38.7)	35.3-42.1	20 (32.3)	29-35.6	
Ex-smoker	16 (20.6)	17.8-23.4	16 (25.4)	22.4-28.4	34 (54)	50.5-57.5	
**BMI**							
Not overweight	158 (40.8)	37.4-44.2	143 (37.0)	33.6-40.4	86 (22.2)	19.3-25.1	**<0.001^a^, ^b^**
Overweight	86 (21.3)	18.4-24.2	120 (29.8)	26.6-33	197 (48.9)	45.4-52.4	
**Abdominal obesity**							
No abdominal obesity	136 (35.4)	32.1-38.7	158 (41.2)	37.8-44.6	90 (23.4)	20.4-26.4	**<0.001^b^**
With abdominal obesity	108 (26.7)	23.6-29.8	104 (25.7)	22.7-28.7	193 (47.6)	44.1-51.1	
**Glycemia**							0.004***
Normal	243 (31.5)	28.3-34.7	258 (33.4)	30.1-36.7	271 (35.1)	31.8-38.4	
Altered	1 (5.6)	4-7.2	5 (27.8)	24.7-30.9	12 (66.7)	63.4-70	

* Pearson’s chi-square.

** Chi-square of Linear Trend.

*** Fisher’s exact test.

BP: blood pressure; BMI: Body Mass Index. 95%CI: 95% Confidence Interval.

^a^Significant result comparing prehypertension and normal BP.

^b^Significant result comparing hypertension and normal BP. N=790.

**Table 2:** Simple binary logistic regression and the respective confidence intervals for the association of arterial hypertension with sociodemographic, labor, and nutritional status variables of farmers living in Espírito Santo, according to sex.

	Male				Female			
			95%CI				95%CI	
Variables	*p* value	OR	Lower	Upper	*p* value	OR	Lower	Upper
**Age group** (years)								
≤30		1				1		
31-40	0.604	1.166	0.652	2.085	**<0.001**	0.225	0.138	0.365
41-50	0.104	1.636	0.905	2.959	0.473	1.159	0.774	1.735
>50	**<0.001**	3.980	2.161	7.330	**0.015**	1.870	1.127	3.102
**Race/color**								
White		1				1		
Not white	0.841	0.939	0.509	1.733	**0.037**	0.480	0.241	0.955
**Schooling** (years)								
≤4		1				1		
5-8	0.185	0.719	0.441	1.171	**<0.001**	0.185	0.100	0.342
>8	**0.002**	0.272	0.117	0.632	**<0.001**	0.212	0.094	0.480
**Possession of land**								
Landowner		1				1		
No landowner	0.371	1.248	0.768	2.030	**0.001**	0.469	0.304	0.723
**Weekly workload** (hours/week)								
≤40		1				1		
>40	**0.022**	0.485	0.262	0.899	**0.001**	0.469	0.304	0.723
**Alcohol consumption**								
Do not consume		1				1		
Consume	0.386	1.201	0.794	1.818	**<0.001**	0.348	0.219	0.553
**Smoking**								
Does not smoke		1				1		
Smokes currently	0.450	0.785	0.419	1.472	0.706	1.333	0.298	5.957
Smoked in the past	**0.002**	2.472	1.379	4.429	0.484	0.600	0.143	2.511
**BMI**								
Not overweight		1				1		
Overweight	**<0.001**	3.239	2.130	4.924	0.445	0.899	0.684	1.181
**Abdominal obesity**								
No abdominal obesity		1				1		
With abdominal obesity	**<0.001**	3.340	2.177	5.123	**0.038**	1.773	0.607	0.985
**Glycemia**								
Normal		1				1		
Altered	**0.011**	4.039	1.376	11.857	0.292	1.800	0.603	5.371

BMI: body mass index. N=790.

**Table 3:** Final models of the multiple logistic regression analysis between arterial hypertension and sociodemographic variables and the nutritional status of farmers (both men and women) living in Espírito Santo.

	Male			
			95%CI	
Variables	*p* value	OR	Lower	Upper
**Age group** (years)				
≤30		1		
31-40	0.126	0.624	0.340	1.142
41-50	0.698	0.882	0.469	1.661
>50	0.091	1.818	0.909	3.633
**Schooling** (years)				
≤4		1		
5-8	0.302	0.755	0.443	1.288
>8	**0.004**	0.257	0.103	0.640
**Weekly workload** (hours/week)				
≤40		1		
>40	**<0.001**	0.333	0.187	0.596
**Smoking**				
Not		1		
Smokes currently	0.062	0.510	0.252	1.035
Smoked in the past	0.607	1.190	0.612	2.314
**BMI**				
Not overweight		1		
Overweight	0.098	1.646	0.913	2.970
**Abdominal obesity**				
No abdominal obesity		1		
With abdominal obesity	**0.039**	1.947	1.034	3.667
**Glycemia**				
Normal		1		
Altered	0.604	1.376	0.412	4.595

	Females			
			95%CI	
Variables	*p* value	OR	Lower	Upper
**Age group** (years)				
≤30		1		
31-40	0.065	0.510	0.249	1.044
41-50	**0.021**	2.260	1.128	4.529
>50	**0.002**	3.413	1.568	7.432
**Race/color**				
White		1		
Not white	0.745	0.868	0.371	2.033
**Schooling** (years)				
≤4		1		
5-8	**0.007**	0.367	0.176	0.763
>8	**0.021**	0.335	0.132	0.851
**Possession of land**				
Landowner		1		
Non-landowner	0.276	0.722	0.401	1.299
**Weekly workload** (hours/week)				
≤40		1		
>40	0.876	1.043	0.617	1.763
**Alcohol consumption**				
Do not consume		1		
Consume	0.096	0.602	0.332	1.093
**Abdominal obesity**				
No abdominal obesity		1		
With abdominal obesity	**0.003**	2.531	1.377	4.653

The variables included in the models were those that presented *p*<0.05 in the simple model. BMI: body mass index. N=790.
